# Incidence and predictors of mortality among traumatic brain injury patients admitted to Amhara region Comprehensive Specialized Hospitals, northwest Ethiopia, 2022

**DOI:** 10.1186/s12873-023-00823-9

**Published:** 2023-05-24

**Authors:** Tiruye Azene Demlie, Mahlet Temesgen Alemu, Mengistu Abebe Messelu, Fasil Wagnew, Enyew Getaneh Mekonen

**Affiliations:** 1 Department of Surgical Nursing, School of Nursing, College of Medicine and Health Sciences, University of Gondar, Gondar, Ethiopia; 2 Department of Nursing, College of Medicine and Health Sciences, Debre Markos University, Debre Markos, Ethiopia; 3 College of Health Sciences, Debre Markos University, Debre Markos, Ethiopia; 4 National Center for Epidemiology and Population Health (NCEPH), College of Health and Medicine, The Australian National University, Canberra, Australia

**Keywords:** Traumatic brain injury, Incidence, Mortality, Ethiopia

## Abstract

**Introduction:**

Traumatic brain injury is a substantial cause of mortality and morbidity with a higher burden in low and middle-income countries due to healthcare systems that are unable to deliver effectively the acute and long-term care the patients require. Besides its burden, there is little information on traumatic brain injury-related mortality in Ethiopia, especially in the region. Therefore, this study aimed to assess the incidence and predictors of mortality among traumatic brain injury patients admitted to comprehensive specialized hospitals in the Amhara region, northwest Ethiopia, 2022.

**Methods:**

An institution-based retrospective follow-up study was conducted among 544 traumatic brain injury patients admitted from January 1, 2021, to December 31, 2021. A simple random sampling method was used. Data were extracted using a pre-tested and structured data abstraction sheet. Data were entered, coded, and cleaned into EPi-info version 7.2.0.1 software and exported to STATA version 14.1 for analysis. The Weibull regression model was fitted to determine the association between time to death and covariates. Variables with a P-value < 0.05 were declared statistically significant.

**Results:**

The overall incidence of mortality among traumatic brain injury patients was 1.23 per 100 person-day observation [95% (CI: 1.0, 1.5)] with a median survival time of 106 (95% CI: 60, 121) days. Age [AHR: 1.08 (95% CI; 1.06, 1.1)], severe traumatic brain injury [AHR: 10 (95% CI; 3.55, 28.2)], moderate traumatic brain injury [AHR: 9.2 (95% CI 2.97, 29)], hypotension [AHR: 6.9 (95% CI; 2.8, 17.1)], coagulopathy [AHR: 2.55 (95% CI: 1.27, 5.1)], hyperthermia [AHR: 2.79 (95% CI; 1.4, 5.5)], and hyperglycemia [AHR: 2.28 (95% CI; 1.13, 4.6)] were positively associated with mortality while undergoing neurosurgery were negatively associated with mortality [AHR: 0.47 (95% CI; 0.27-0 0.82)].

**Conclusion:**

The overall incidence of mortality was found to be high. Age, severe and moderate traumatic brain injury, hypotension at admission, coagulopathy, presence of associated aspiration pneumonia, undergoing a neurosurgical procedure, episode of hyperthermia, and hyperglycemia during hospitalization were the independent predictors of time to death. Therefore, interventions to reduce mortality should focus on the prevention of primary injury and secondary brain injury.

## Introduction

Head injury can be defined as a definite history of an upset to the head, a laceration of the scalp, or altered consciousness secondary to physical injury/structural alteration to the skull by any type of external force to the head [1]. Traumatic brain injury (TBI) is a non-congenital injury to the brain from an external mechanical force that leads to permanent or temporary impairment of cognitive, physical, and psychosocial functions [2, 3]. Globally, TBI is a considerable cause of mortality and morbidity across all age groups, with a greater burden in low and middle-income countries (LMICs) due to the high prevalence of risk factors and the inability of health systems to deliver acute and long-term care effectively [4–6]. The incidence of TBI worldwide is rising, mainly owing to injuries associated with the increased use of motor vehicles, particularly in developing countries [7].

The World Health Organization (WHO) global burden of injury estimation ranks injury among the top ten leading causes of death worldwide, of these TBI, is the leading cause of death and disability accounting for about 30% of all injury-related deaths [4, 6, 8, 9]. Current estimates suggest that about 4.48 million people died due to traumas which accounts for 8% of all deaths globally, 38% more than the number of deaths from malaria, tuberculosis, and HIV/AIDS combined [8]. Of these, an estimated 2 million deaths were attributed to the TBI [2, 11], and the burden was concentrated in LMICs due to limited access to advanced life-sustaining measures after trauma [12].

In the United States of America (USA), TBI is the commonest cause of mortality and disabilities [10]. Annually, more than 2.8 million TBI cases were recorded with a 2% of mortality [11, 12]. In Africa, TBI is a hidden epidemic, one-third of all head injury patients suffer from poor outcomes, and those patients with severe head injury have almost twice the risk of dying compared to those in high-income countries [13, 14]. The resource constraints and inconsistencies in clinical personnel training play an important role when considering the treatment of patients with TBI [15]. Given these problems, the probability of dying from TBI is far greater in LMICs, including Ethiopia, with some areas experiencing as high as a 50% mortality rate in moderate-to-severe cases of TBI [19].

Studies suggested that early prevention of TBI is important to save lives, minimize disabilities and reduce healthcare-related costs [16]. Among these prevention of secondary brain injury plays a major role in the reduction of mortality, the common reason behind secondary brain injury is increased intracranial pressure (ICP), if appropriate treatments to maintain ICP level within the normal limit are not initiated timely, brain herniation can occur and lead to death [17].

In Ethiopia, TBI is a significant public health problem with a reported prevalence of 39.7% and it is the leading reason behind mortality and disability [1, 18]. The effects of TBI are not limited to an individual’s health but are also a cause of increased socioeconomic burden to the family as well as to the country in general [19]. The incidence of mortality secondary to TBI is ranging from 2.26 to 2.6 per 100-person day observations in Ethiopia [5] [14]. The federal government of Ethiopia has proclaimed the rules and regulations about prevention strategies, but despite the measures taken so far still mortality and severe disability were significantly associated with TBI [20]. Thus, a thorough understanding of TBI in LMICs including Ethiopia is essential to mitigate TBI-related mortality [15]. Therefore, this study aimed to assess the incidence and predictors of mortality among TBI patients admitted to comprehensive specialized hospitals in the Amhara region.

## Methods

### Study design and period

A multicenter retrospective follow-up study was employed from Jan.1, 2021, to Dec.31, 2021, and data was extracted from May 15 – June 15/2022.

### Study setting

The study was conducted in comprehensive specialized hospitals in the Amhara regional state of Northwest Ethiopia. Amhara region is one of the 12 regional states which is located in the northwestern part of Ethiopia with an estimated area of 159,173.66 square kilometers. The region is administratively organized into 12 zones, three-city administrations, and 183 districts. According to the 2020 Ethiopian fiscal year report. The total population projection of the region is estimated at 22,191,890 (11,317,864 males and 10,874,026 females) and according to Amhara National Regional Health Bureau, based on the Annual Performance Report, the region has 81 Hospitals, 858 Health Centers, and 3560 Health Posts. Among those 81 hospitals in the region, there are 8 comprehensive specialized hospitals. Of these, the University of Gondar, Felege-Hiwot, Tibebe-Ghion, Debre-Markos, and Deberetabor are comprehensive specialized hospitals found in northwest Amhara. Thus, all those five comprehensive specialized hospitals serve the population found in the area [21]. Those five hospitals served approximately 22,127 adult trauma cases a year, out of the total adult trauma cases TBI is estimated to be 5700 / year.

### Source populations

All adult patients admitted with traumatic brain injury in Amhara regional state comprehensive specialized hospitals in northwest Ethiopia.

### Study populations

Adult TBI patients who were admitted to Amhara regional state comprehensive specialized hospitals from January 1, 2021, to December 31, 2021.

### Inclusion and exclusion criteria

All records of adult TBI patients admitted to the comprehensive specialized hospitals of the Amhara region from January 1, 2021, to December 31, 2021, were included in the study. Incomplete charts missed the outcome record, and patients who were transferred in from other institutions were excluded from the study.

### Sample size determination and sampling procedure

The sample size was calculated using single population proportion formula by taking the estimated incidence of TBI mortality at 30.4% from a previous study conducted at Felege Hiwot CSH [14]. 95% confidence interval and 4% margin of error. By adding a 10% for possible incomplete charts, the final sample size was 563. The patient chart was selected by simple random sampling, using a computer-generated table of random numbers.

### Variables of the study

Incidence of mortality is the dependent variable.

Independent Variables: Socio-demographic factors (age, sex, and residence); injury-related factors (mechanism of injury, type of injury, the occurrence of Co-existing injury); clinical and diagnostic factors (oxygen saturation, pupillary reflex, Admission GCS, Blood pressure, Blood sugar level, CT scan, comorbidity, hemoglobin level, coagulopathy, and temperature); and management-related factors: (Neurosurgery, Mannitol, phenytoin, intubation, and mechanical ventilation)

### Operational definitions

#### Censored

TBI patients survive at the end of the follow-up period, are lost to follow-up, transferred to a different institution, or left against medical advice.

#### Event

Occurrence of death from the first confirmed diagnosis of traumatic brain injury until the end of the follow-up.

#### Time to death

Calculated as the number of days between the date of diagnosis of TBI and the date of death.

**Mild TBI**:- GCS: 13–15; **Moderate TBI**:-GCS: 9–12; **Severe TBI**:- GCS: ≤8 [22].

#### Type of injury

Is coded as penetrating and blunt based on skin integrity [23].

#### Poly-trauma

Is defined as the trauma of more than two anatomical areas [24].

**Hypoglycemia** is defined as a random blood glucose concentration level of < 80 mg/dl during hospitalization [25], and hyperglycemia is defined as a random blood sugar level of ≥ 200 mg/dl [26].

### Data collection tool and procedure

Data were extracted by using an appropriate data extraction tool by reviewing patient charts. The data extraction sheet was developed in the English language. It contains four socio-demographic-related items, nineteen clinical and radiologic-related items, six injury-related items, and four management-related items with a total of 33 questions.

### Data quality control

To ensure the quality of data, the data extraction tool was checked for the existence of variables in the registration format on the patient’s chart via a preliminary chart review of 10 charts at UOGCSH, thus appropriate modifications were made after analyzing the pretest result before the actual data collection. Face validity was done by emergency and critical care experts. On-site Training was given to data collectors and supervisors on data collection tools and procedures for one day for each site. Data collectors were supervised closely by supervisors. The completeness of each abstraction sheet was checked by the principal investigator and supervisors on a daily base.

### Data processing and analysis

Data were cleaned, coded, and entered, into EPi-info version 7.2 software and exported to STATA version 14.1 statistical software. Descriptive statistics were expressed by a median with Interquartile Range whereas categorical variables were expressed by the frequency with percentage. The outcome of each participant was dichotomized into censored or event. The Incidence Density Rate of mortality was calculated for the entire follow-up period. Kaplan Meier (KM) survival curve was used to estimate the median survival time and cumulative probability of survival and the KM failure curve together with the log-rank test was fitted to test the presence of difference in the probability of death among the groups. Both bi-variable and multivariable Weibull regression was used to identify predictor variables. Variables having a p-value < 0.25 in the bi-variable analysis were entered into the multivariable analysis and an Adjusted Hazard Ratio (AHR) with 95% Confidence Intervals (CI) was computed to evaluate the strength of association and variables with a p-value less than 0.05 were considered as statistically significant.

## Results

### Socio-demographic and injury-related factors

A total of 563 records of TBI patients were reviewed. Of these, 544 (96.6%) charts were included in the analysis. The majority of patients were male 466 (85.6%). Three hundred ninety-three (72.24%) of patients were between 18 and 40 years, and 27 (4.96%) patients were above 65 years with a median age of 32 years (IQR: 25–42 years). More than two-thirds of the patients 373(68.57%) were rural residents. Assaults were the leading cause of TBI 327 (60.11%) followed by road traffic incidents 138 (25.37%). One-third of patients, 164 (30.15%) suffered multiple injuries to the chest, face, extremities, abdomen, and pelvis. Of the total patients, 95(17.46%) had associated aspiration pneumonia. The overall median length of stay was 7 days (IQR: 3–14) (Table 1). Clinical and Management-related findings at admission, the median GCS was 13 (IQR, 10–14). Two hundred ninety -five (54.23%), 166 (30.1%), and 83 (15.26%) patients had sustained mild, moderate, and severe TBI respectively. At admission, 21 (3.86%) were hypotensive, 42 (7.72%) were hypoxic, and two-thirds of patients, 352 (64.71%) had a brain CT scan done. Of these, 127 (36.08%) were contusions followed by subdural hematoma 98, (27.84%). Regarding the pupillary reactivity to light, 46 (8.46%) of patients presented with bilateral non-reactive pupils, and 64 (11.76%) had unilateral reactive pupils. Two hundred thirty-five (43.2%), and 441 (81.07%) patients had received mannitol and phenytoin as prophylaxis of increased ICP and anti-seizure, respectively. Among the cohort, 247 (45.4%) of the patients had undergone neurosurgery. One hundred thirty-four (54%) of patients underwent a craniotomy (Table 2).

**Table 1:** Socio-demographic and injury-related characteristics of TBI patients admitted in Amhara Region, comprehensive specialized hospitals, 2022 (n = 544)

Variables	Category	Total (%)	Death (%)	Censored (%)	PDO	Incidence density
Age	18–40 years	393(72.2)	33(8.4)	360(91.6)	5103	0.0065
	41–64 years	124(22.8)	32(25.81)	92(74.19)	1283	0.025
	>=65 years	27(4.96)	16(59.26)	11(40.74)	178	0.089
Sex	Male	466(85.6)	71(15.24)	395(84.76)	5949	0.012
	Female	78(14.4)	10(12.82)	68(87.18)	615	0.016
Residence	Urban	171(34.3)	20(11.7)	151(88.3)	2429	0.008
	Rural	373(65.7)	61(16.31)	312(83.65)	4135	0.015
Mechanism of injury	RTA	138(25.3)	41(29.71)	97(70.29)	1464	0.028
	Assault	327(60.1)	22(6.73)	305(93.27)	4060	0.005
	Fall	75(13.8)	17(22.67)	58(77.33)	851	0.019
	Other*	4(0.74)	1(25)	3(75)	189	0.005
Type of head injury	Blunt	131(24.1)	18(13.74)	113(86.26)	1083	0.016
	Penetrating	413(75.9)	63(15.25)	350(84.75)	5481	0.011
Presence of coexisting trauma	Yes	164(30.2)	52(31.71)	112(68.29)	2122	0.025
	No	380(69.8)	29(7.63)	351(92.37)	4442	0.006
Type of coexisting trauma(n = 164)	Maxillofacial	66(40.2)	17(25.76)	49(74.24)	898	0.019
	Chest injury	32(19.5)	15(46.88)	17(53.13)	564	0.026
	Abdo. injury	5(3.1)	0	5(100)	34	0
	Pelvic injury	10(6.1)	1(10)	9(90)	105	0.0095
	Poly-trauma	51(31.1)	19(37.25)	32(62.75)	521	0.036
Aspiration pneumonia	Yes	95(17.5)	53(55.79)	42(44.21)	1411	0.037
	No	449(82.5)	28(6. 24)	421(93.76)	5153	0.005

**Note**: RTA = road traffic accidents; PDO: person, day, observation; *burn, animal bite

**Table 2:** Clinical and management-related characteristics of TBI patients admitted in Amhara Region, comprehensive specialized hospitals, 2022

Variables	Category	Total (%)	Survival status		PDO	Incidence density
			Death (%)	Censored (%)		
Hypotension	Absent	523(96.1)	69(13.2)	454(86.8)	6362	0.011
	Present	21(3.9)	12(57.14)	9(42.86)	202	0.059
Hypoxia	Yes	42(7.7)	32(76.19)	10(23.81)	574	0.055
	No	502(92.2)	49(9.76)	453(90.24)	5990	0.008
Severity of TBI based on admission GCS	Mild TBI	295(54.2)	6(2.03)	289(97.97)	3159	0.002
	Moderate TBI	166(30.5)	16(9.64)	150(90.36)	1796	0.009
	Severe TBI	83(15.3)	59(71.08)	24(28.92)	1609	0.036
Pupillary reactivity to light	Both reactive	434(79.8)	23(5.3)	411(94.7)	4670	0.005
	Unilateral reactive	64(11.7)	22(34.38)	42(65.63)	835	0.026
	Both nonreactive	46(8.5)	36(78.26)	10(21.74)	1059	0.034
Hypoglycemia	Absent	510(93.7)	54(10.59)	456(89.41)	5893	0.009
	Present	34(6.3)	27(79.41)	7(20.59)	671	0.04
Hyperglycemia	Absent	433(79.6)	46(10.62)	387(89.38)	4860	0.009
	Present	111(20.4)	35(31.53)	76(68.47)	1704	0.020
Hypothermia	Absent	441(81)	53(12.02)	388(87.98)	4607	0.012
	Present	103(19)	28(27.18)	75(72.82)	1957	0.014
Fever	Absent	323(59.4)	17(5.26)	306(94.74)	3882	0.004
	Present	221(40.6)	64(28.26)	157(71.04)	2682	0.023
Seizure	Yes	65(22)	24(36.92)	41(63.08)	721	0.033
	No	479(88)	57(11.9)	422(88.1)	5843	0.01
CT scan	Yes	344(63.2)	47(13.66)	297(84.38)	3892	0.012
	No	200(36.8)	34(17)	166(83)	2672	0.013
Ct finding (N = 344)	Brain contusion	125(36.3)	9(7.2)	116(92.8)	1327	0.007
	Subdural hematoma	97(28.2)	16(16.5)	81(83.5)	1114	0.014
	Epidural hematoma	62(18)	5(8)	57(92)	693	0.007
	DAI	35(10)	17(49.6)	18(51.4)	483	0.035
	Others **	25(7.3)	0	25(100)	275	0
Comorbidity	Yes	15(2.8)	6(40)	9(60)	146	0.041
	No	529(97.2)	75(14.18)	454(85.82)	6418	0.012
Mannitol	Yes	235(43.2)	67(28.5)	168(71.5)	3519	0.019
	No	309(56.8)	14(4.53)	295(95.47)	3045	0.005
Neurosurgery	Yes	247(45.4)	24(9.72)	223(90.28)	3408	0.007
	No	297(54.6)	57(19.19)	240(80.81)	3156	0.018
Type of neurosurgery (N = 247)	Craniotomy	134(54)	17(12.69)	117(87.31)	1680	0.01
	Burrhole	42(16.9)	7(16.67)	35(83.33)	718	0.01
	Elevation	65(26.9)	0	65(100)	938	0
	Other*	7(2.8)	1(14.29)	6(85.71)	77	0.013

GCS: Glasgow coma scale,*craniectomy, toilet surgery, debridement, and duraplasty

DAI: diffused axonal injury ** depressed skull fracture, pneumocephalus, subarachnoid hemorrhage

### Incidence of mortality

**Fig. 1:**
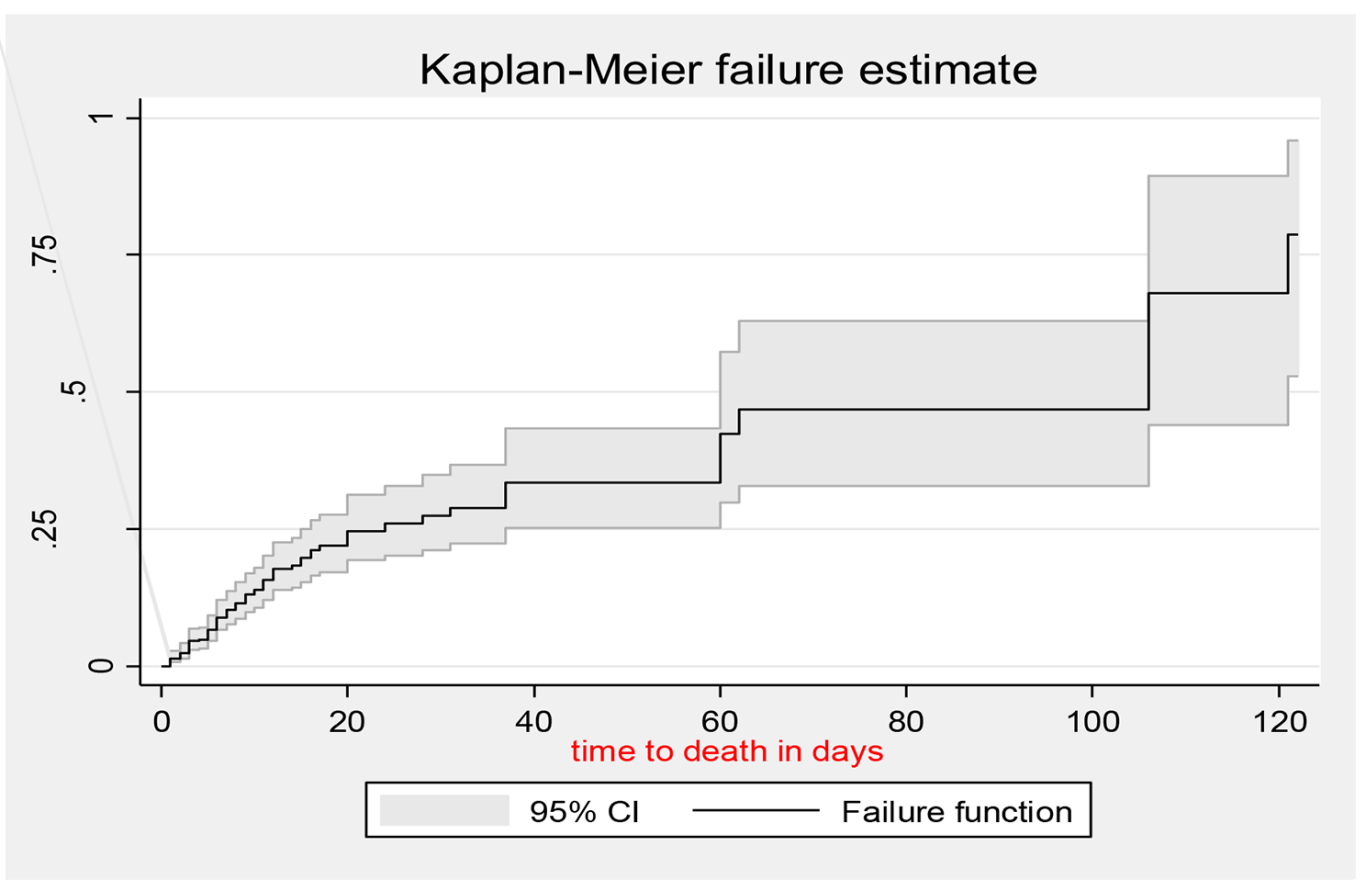
Overall Kaplan-Meier failure estimation of admitted TBI patients in the Amhara region, 2022

**Fig. 2:**
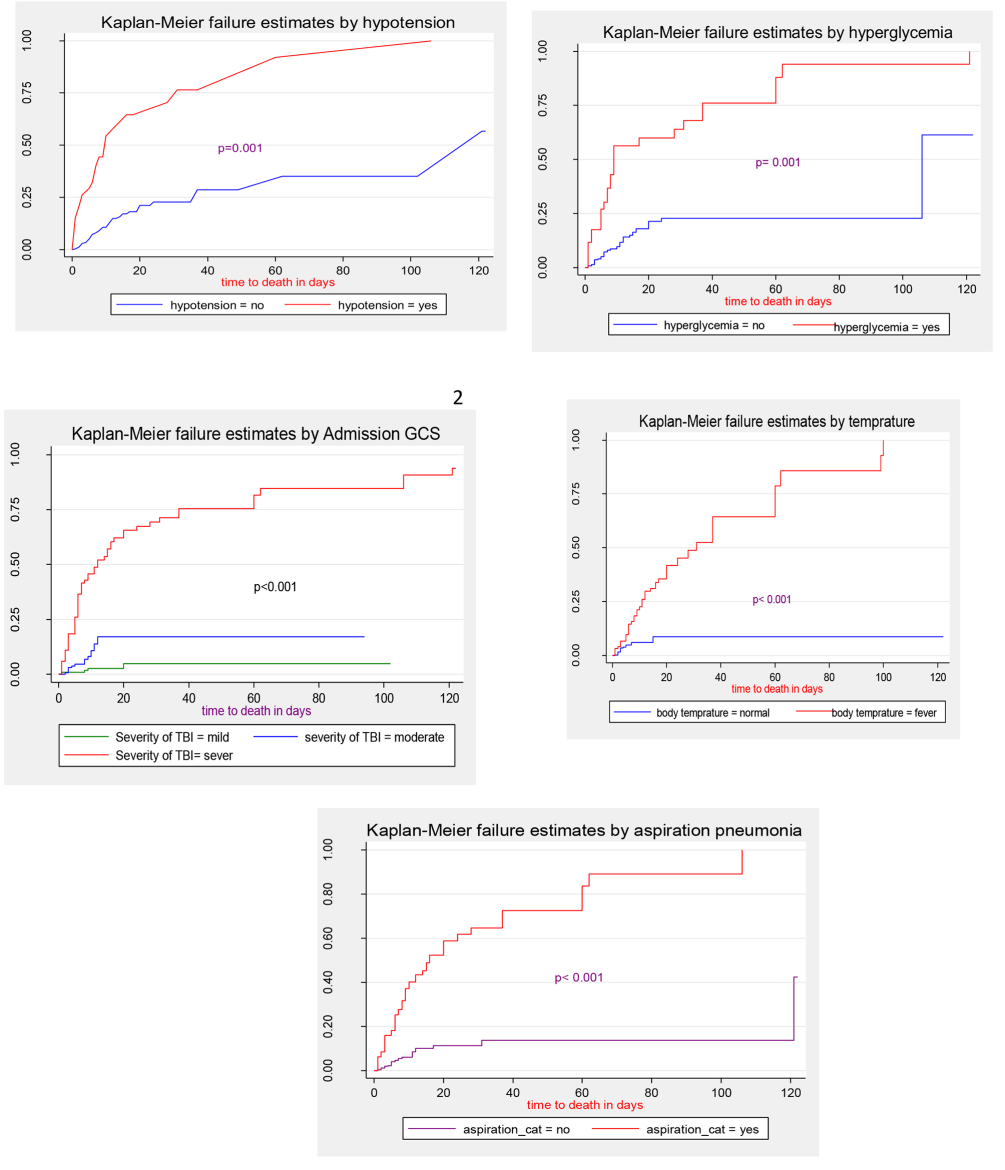
Kaplan-Meier failure estimate for hypotension, hyperglycemia, Aspiration pneumonia, GCS, and temperature among TBI patients admitted in the Amhara region, 2022

The total time at risk for 544 patients was 6,564 person-days with an incidence rate of 1.23 (95% CI: 1.0–1.5) per 100-person day observation. The maximum number of days of follow-up was 120 days. The overall median survival time was 106 (95% CI: 60–121) days (Fig. 1). Kaplan–Meier failure curve together with the log-rank test was fitted to test for the presence of a difference in the occurrence of death among the categorical variables. The incidence of mortality in patients with severe TBI was 3.66 per 100 person-days it was 0.9 and 0.2 per 100 person-days for moderate and mild TBI, respectively. At seven days of follow-up, the probability of survival for those who sustained mild, moderate, and severe TBI was 96.7%, 82.9%, and 48.1%, respectively. The incidence of mortality was higher among hypotensive patients (Fig. 2).

### Factors associated with the incidence of mortality

In the bi-variable analysis of baseline clinical and management-related variables (initial GCS, admission systolic BP *<* 90 mmHg, hypoxia and neurosurgery), age, residence, type of head injury presence of extra-cranial injuries, presence of comorbidity, pupillary reactivity abnormality, episode of hyperglycemia, episode of seizure, presence of aspiration pneumonia, coagulopathy and an episode of hyperthermia, were showed association with time to death at a p-value *<* 0.25. However, in the final multivariable Weibull regression model; age, initial GCS, admission BP, neurosurgery, episode of hyperglycemia, coagulopathy, presence of associated aspiration pneumonia, type of head injury, and episode hyperthermia were found to be independent predictors of mortality. The hazard of death among patients who underwent neurosurgery was 53% (AHR: 0.47; 95% CI; 0.27-0 0.82) times lower than those who did not undergo neurosurgery. The hazard of death among patients with severe and moderate TBI was 10(AHR: 10; 95% CI; 3.5–28.2) and 9.2 times (AHR: 9.2; [95% CI 2.97-29]) higher than those with mild TBI, respectively. As age increases by one year, the hazard of death among TBI patients increased by 8% (AHR: 1.08; 95% CI; 1.06–1.1). The hazard of death among hypotensive patients was 6.9 (AHR: 6.9 [95% CI; 2.8–17.1]) times higher than those with normotensive. The hazard of death among hyperglycemic patients was 2.28 (AHR: 2.28; [95% CI; 1.13–4.6]) times higher than those with normoglycemic. The hazard of death among hyperthermic patients was 2.79 [AHR: 2.79; [95% CI; 1.4–5.5]) times higher than normothermic patients. The hazard of death among coagulopathic patients was 2.48 (AHR: 2.48; [95%CI 1.19–5.17]) higher than in patients that do not develop coagulopathy (Table 3).

**Table 3:** Bi-variable and multivariable Weibull regression analysis for independent predictors of time to death among TBI patients admitted in n Amhara region, 2022

Variables	Category	Survival status		CHR(95%CI)	AHR(95%CI)	P-value
		Death	Censored			
Age in years	Cont.	81(14.8)	463(85.1)	1.06(1.04–1.07)	1.08(1.06–1.1)	< 0.001^*^
Residence	Urban	20(11.7)	151(88.3)	1	1	1
	Rural	61(16.35)	312(83.65)	1.81(1.08-3).	1.05(0.59–1.88)	0.847
Extra-cranial injury	Present	52(31.71)	112(68.29)	3.9(2.5–6.4)	1(0.58–1.73)	0.985
	Absent	29(7.63)	351(92.37)	1	1	1
Type of head injury	Blunt	18(13.74)	113(86.26)	1.4(0.82–2.4)	3.2(1.5–6.8)	0.002^*^
	Penetrating	63(15.25)	350(84.75)	1	1	1
The severity of the head injury	Mild	6(2.03))	289(97.97)	1	1	1
	Moderate	16(9.64)	150(90.36)	4.5(1.8–11.6)	9.2(2.97-29)	< 0.001*
	Sever	59(71.08)	24(28.92)	23(10–54)	10(3.6–28.2)	< 0.001*
Pupillary reactivity	Bilateral reactive	23(5.3%)	411(94.7%)	1	1	1
	Unilateral reactive	22(34.38)	42(65.63%)	5.6(3.1–10)	0.88(0.42–1.85)	0.749
	Both non-reactive	36(78.26)	10(21.74)	8.9(5.2–15)	0.74(0.33–1.65)	0.466
Seizure	Yes	24(36.92)	41(63.08)	3.9(2.9–6.4)	1.15(0.51–2.57)	0.733
	No	57(11.9)	422(88.1)	1	1	1
Hypoxia	Yes	32(76.19)	10(23.81)	6.6(4.2–10)	1.46(0.79–2.72)	0.223
	No	49(9.76)	453(90.24)	1	1	1
Comorbidity	Yes	6(40)	9(60)	3.4(1.5–7.8)	0.36(0.11–1.13)	0.081
	No	75(14.18)	454(85.82)	1	1	1
Coagulopathy	Yes	21(52.5)	19(47.5)	2.5(1.5–4.15)	2.48(1.19–5.17)	0.015
	No	60(11.9)	444(88.1)	1	1	1
Aspiration pneumonia	Yes	53(55.79)	42(44.21)	7.5(4.7–11.8)	3.2(1.76–5.9)	< 0.001*
	No	28(6.24)	421(93.76)	1	1	1
Fever	Yes	64(28.26)	157(71.04)	5.7(3.3–9.7)	2.79(1.4–5.5)	0.004^*^
	No	17(5.26)	306(94.74)	1	1	1
Hypotension	Yes	24(61.5)	15(38.5)	5.04(3.1–8.1)	6.9(2.8–17.1)	< 0.001*
	No	57(11.3)	448(87.7)	1	1	1
Hyperglycemia	No	46(10.62)	387(89.38)	1	1	1
	Yes	35(31.53)	76(68.47)	2.3(1.5–3.7)	2.28(1.13–4.6)	0.020^*^
Neurosurgery	Yes	24(9.72)	223(90.28)	0.4(0.25–0.65)	0.47(0.27-0 0.82)	0.008 ^*^
	No	57(19.19)	240(80.81)	1	1	1

**Fig. 3:**
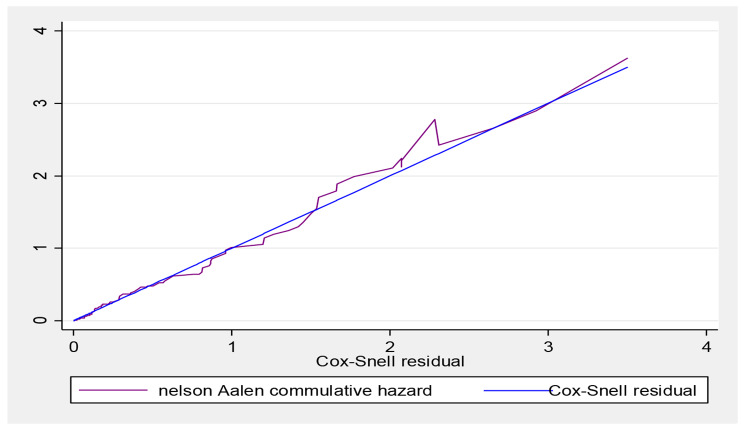
Nelson-Aalen cumulative hazard graph against Cox-Snell residual on TBI patients admitted in Amhara region, 2022

Proportional hazard assumptions were checked both graphically by using a log (-log) plot for each predictor variable and statistically using a Schoenfeld residual test and satisfied at (p**-**value = 0.9320). Log likely hood and Akaike Information Criteria (AIC) were applied to select the best-fitted model, based on this the Weibull regression was selected **(**Table 4**)**. The goodness of fit test of the final model was checked by using Cox Snell residuals against the Nelson-Aalen cumulative hazard function. The hazard function follows 45˚ close to the baseline hazard which indicated that the model was well-fitted. For the residual test, it was possible to conclude that the final model fitted well (Fig. 3).

**Table 4:** Summary of model comparison based on Akaike information criteria

Model	Observation	Log likely Hood	DF	AIC	BIC
Weibull	544	-154.72	19	347.44	441.12
Exponential	544	-159.94	18	355.88	433.26
Gompertz	544	-159.93	19	357.86	439.54
Cox	544	-313.13	17	660.26	733.34

## Discussion

TBI is a leading cause of mortality and disability worldwide accounting for about 30% of all injury-related deaths. The overall incidence rate of mortality among TBI patients was found 1.23 (95%CI: 1.0-1.5) per 100 persons, day observation. This finding is lower than studies conducted in Hawasa university hospital’s 2.26 per 100-person day observation [5] and Felegehiwot comprehensive specialized hospital’s 25.53 per 1000-person day observation [14]. This discrepancy could be differences in time and variations in duration of follow-up in this research the total time at risk was 6544 person-days while it was 6,542 person-days and 4032 person-days for Hawasa and Felegehiwot respectively. This study found that 14.89% [95% CI (12.13–18.15%)] of patients died during the follow-up. This finding is in line with a study conducted at Hawasa University hospital12.7% [5]. But this result is lower than studies conducted in Jimma University Specialized Hospital21%[4], India 34.58% [2], Felegehiwot Comprehensive Specialized Hospital30.4%[14], and Qatar27% [27]. This possible discrepancy could be because there is a time difference which will lead to increased access to health institutions, increased awareness about early health-seeking behavior, and advancements in neurosurgical interventions. However, this result is higher than studies conducted in Tikur Anbesa specialized hospital10.3%[3] and China 5%[28]. This possible difference may be poor nursing interventions and a lack of advanced life support after trauma in our setting [29]. Delays in the care of patients with severe head injuries lead to worse outcomes both in terms of survival and functional status. The problem is compounded by a few hospitals providing neurosurgical services in the entire region. This has created even more delays to the optimum care that the patients can get [30]. And also the study period of this study was high in a conflict which may increase trauma admission and may further increase delays to the optimum care that the patients can get. The current finding revealed that age is a predictor of mortality following TBI. This result is supported by studies conducted in Canada [31], Tanzania [32, 33], Rwanda [34], Qatar [27], Hawasa [5], Tikur Anbesa [3], and Felegehiwot comprehensive specialized hospitals [14]. The reason behind this increased mortality could be older age patients have less immunity leading to infection and are also less likely to undergo surgery due to fear of complications related to anesthesia. Also, various cellular and molecular changes occur in the constituents of the brain during the aging process [35]. There is also white matter shrinkage and decreased myelination in the aging brain, which is essential for rapid, integrated neuronal communication [36]. Among injury-related factors, this study found that blunt TBI^s^ have increased the death hazard as compared to penetrating TBIs. This result is in agreement with a study conducted in Uganda [37]. However, this result in on contrary to a study conducted in Texas America [38]. This possible difference could be due to giving less attention to blunt injuries in our setting. Tissue swelling from a traumatic brain injury can increase pressure inside the skull and cause additional damage to the brain leading to ischemia and necrosis [39]. Based on this study, the severity of TBI based on admission GCS was a strong predictor of mortality. This finding is parallel with studies conducted in different countries like Qatar [27], Uganda [37], Tanzania [32], Rwanda [34], Sub-Saharan Africa [15], Hawassa University Hospital [5], TASH [3], and Felegehiwot comprehensive specialized hospital [14]. The possible reason could be those patients with lower GCS scores are unable to protect their airways and they are at risk of aspiration, have poor infection prevention, are not a candidate for neurosurgery, and have less access to intensive care unit settings [40]. This research found that neurosurgical interventions are associated with the greatest decrement in the hazard of death among TBI patients. This finding is supported by different literature conducted in Tanzania [33], Spain [41], Uganda [37], Hawasa [5], and Felegehiwot [14]. This is due to bleeding outside or within the brain can result in a collection of clotted blood (hematoma) that puts pressure on the brain and damages brain tissue, thus surgery relieves pressure inside the skull by draining accumulated blood or creating a window in the skull that provides more room for swollen tissues [19].This study found that Coagulopathy patients have an increased hazard of death. This result is supported by a study conducted in America [38], and China [42]. This is because following trauma there is a massive release of tissue factor, altered protein C homeostasis, micro-particle up-regulation, and platelet hyperactivity which results in dysfunction and severely compromised hemostatic performance leading to death [43]. Hyperglycemia was an independent predictor of mortality following TBI. This finding is supported by a study done in Iran [44] and China [28]. This is true that following damage and stress catechol amines increase glucagon secretion and inhibit insulin secretion this will lead to the development of hyperglycemia which contributes to morbidity and mortality via generating a noxious cellular environment, causing electrolyte irregularities, and depressing immune efficacy [45]. On the other hand, hypotensive patients were at greatest risk of death hazard as compared to normotensive patients. This finding is in line with a study conducted at Hawasa university hospital [5].cerebral perfusion is directly proportional to mean arterial pressure so being hypotensive leads to decreased cerebral perfusion in addition to increased ICP leads to brain hypoxia and necrosis [46]. This study showed that being hyperthermic increases the death hazard as compared to normothermic patients. This result is parallel with a study conducted at Hawasa University hospital [5] and Felegehiwot comprehensive specialized hospital [14]. This is because as the patient becomes febrile metabolism in general increases and minor changes in brain temperature can result in significant changes in neural cell metabolism and therefore in brain function [47]. The presence of aspiration pneumonia was also positively associated with TBI mortality. This finding is supported by a study conducted in Felegehiwot comprehensive specialized hospital [14]. The entry of fluid into the bronchi and alveolar space triggers an anti-inflammatory reaction with the release of pro-inflammatory cytokines, tumor necrosis factor-alpha, and interleukins, which results in the infectious process [48].

## Conclusion

The incidence of mortality rate among TBI patients was high. Age, moderate and severe TBI, hypotension at admission, coagulopathy, presence of associated aspiration pneumonia, undergoing the neurosurgical procedure, episode of hyperthermia, and hyperglycemia during hospitalization was the independent predictors of mortality among TBI patients. Therefore, interventions to reduce mortality should focus on the prevention of primary Injury and secondary brain injury by giving special emphasis to the patient with vital sign derangement, and following patients’ vital signs attentively. Better to take care of fluid management and better to give special attention to patient prioritization for neurosurgery.

## Data Availability

All data will be available upon a reasonable request. The reader could contact the corresponding author for the underlying data.

## Abbreviations

AHR: Adjusted Hazard Ratio
CHR: Crude Hazard Ratio
CI: Confidence Interval
GCS: Glasgow Coma Scale
LMICs: Low and Middle-Income Countries
RTA: Road Traffic Accident
TBI: Traumatic Brain Injury
UOGCSH: University of Gondar Comprehensive Specialized Hospital
USA: United States of America
WHO: World Health Organization
